# An integrated EMD adaptive threshold denoising method for reduction of noise in ECG

**DOI:** 10.1371/journal.pone.0235330

**Published:** 2020-07-15

**Authors:** Miao Zhang, Guo Wei

**Affiliations:** School of Electrical Engineering and Automation, Harbin Institute of Technology, Harbin, China; Victoria University, AUSTRALIA

## Abstract

Electrocardiogram (ECG) denoising is a biomedical research area of great importance. In this paper, an integrated empirical mode decomposition adaptive threshold denoising method (IEMD-ATD) is proposed for processing ECGs. Three methods are included in the IEMD-ATD. First, an integrated EMD method based on a framework of complete ensemble empirical mode decomposition with adaptive noise (CEEMDAN) is proposed to improve the decomposition quality and stability of raw ECGs. Second, a new grouping method for intrinsic mode functions (IMFs) is developed based on the energy and eigenperiod of IMFs. The grouping method is able to determine the boundaries among high-frequency noise predominant IMFs, useful information predominant IMFs and IMFs with low-frequency noises. Finally, an adaptive threshold denoising method is derived and used for denoising high-frequency noise predominant IMFs. There are two main contributions: 1) an adaptive threshold determination method based on the 3σ criterion and 2) a peak filtering denoising method for retaining useful information contained in the values smaller than the threshold. Synthetic and real ECG data in the MIT-BIH database are utilised in experiments to illustrate the effectiveness of IEMD-ATD for ECG denoising. The results indicate that IEMD-ATD offers better performance in improving the signal-to-noise ratio (SNR) and correlation coefficient compared with the existing EMD denoising methods. Our method offers obvious advantages, especially in retaining detailed information on the QRS complex of the ECG, which is significant for the feature extraction of ECG signals and for pathological diagnosis.

## 1. Introduction

An electrocardiogram (ECG) is a recording of physiological electrical signals produced by human cardiac activity. It can reflect the health state of the heart and is of great importance for diagnosing cardiac diseases such as arrhythmia and ventricular premature beat. However, raw ECG signals are inevitably contaminated by categories of noise such as electromyogram (EMG) noise, baseline wandering, power line interference, electrode contact noise, motion artefacts and thermal noise due to the interference of muscle activities, respiratory movements and ECG acquisition equipment. This noise contaminates the details of ECG morphology, such as P and T waves, which may lead to misjudgements. Therefore, noise reduction for ECGs is very important and necessary.

To eliminate noise from ECG signals effectively, researchers have proposed various algorithms, including classic digital filters based on Fourier analysis, adaptive filters [1–3], neural networks [4], modern statistical techniques, and wavelet denoising algorithms [5, 6]. Since Huang et al. first proposed empirical mode decomposition (EMD) [7] in 1998 for analysing nonlinear and nonstationary signals, numerous noise reduction methods for ECGs based on EMD have been subsequently proposed [8–14].

In [15, 16], Abdel O. Boudraa et al. applied the wavelet soft and hard thresholding method directly to EMD denoising research. The denoising of several typical signals, including the ECG signal, was studied. Compared with median filtering and wavelet denoising, the EMD denoising method offers better performance. Tang Jing-tian et al. combined EMD with wavelet soft and hard thresholding methods in [17] to analyse the denoising effect of ECG signals in detail. They concluded that the EMD denoising results were superior to wavelet denoising in terms of the reconstructed signals. Md. Ashfanoor Kabir and Celia Shahnaz [8] denoised ECG signals by combining EMD and wavelet analysis. First, noisy ECG signals were decomposed by EMD. Then, the first three orders of intrinsic mode functions (IMFs) were superimposed, and windowing was performed to retain the information of the QRS complex. Finally, the EMD-enhanced ECG signal was further denoised by the wavelet soft thresholding method to improve the performance of ECG denoising. Wahiba Mohguen et al. [13] proposed an improved universal threshold and a custom threshold function based on EMD and noise decomposition characteristics. However, the method was reasonable only as an EMD denoising method.

The major problems of the ECG denoising methods based on EMD mentioned above are as follows: 1) EMD is prone to the mode mixing problem when it decomposes ECG signals. Mode mixing has a great influence on ECG denoising, which is the main shortcoming of the EMD denoising method [18]. 2) For the existing IMF grouping methods, the principles are expounded unclearly, and there is no quantitative expression. 3) Although there are many threshold determination methods, none are suitable for various situations. 4) Hard, soft and interval threshold denoising methods will lose local useful information, while other local windowing denoising methods are too complex and ill-suited for application.

To avoid the mode mixing problem, ensemble empirical mode decomposition (EEMD) was proposed [19], which is based on the concept of auxiliary noise. However, the introduction of auxiliary noise leads to new problems, such as more residual noise in the IMFs and more errors in the reconstructed signals. Torres et al. proposed complete ensemble empirical mode decomposition with adaptive noise (CEEMDAN) in [20]. It is an improved method in the EMD family. CEEMDAN preserves the advantage of EEMD in that mode mixing is suppressed. Benefiting from a unique framework, the problem of different numbers of IMFs caused by assisting noises in EEMD is solved, and perfect reconstruction is realised. Nevertheless, CEEMDAN is subject to spurious modes [21]. Additionally, due to the extra procedures required for decomposing white Gaussian noise, the internal calculation times of CEEMDAN increase to a large extent. In addition, the residual noise in each IMF still exists.

Various threshold determination methods have been proposed, such as Universal [22], Heuristic, Minimax, SURE, and EMD Custom Threshold [13]. Since there are various noise types in ECGs, the interaction results of these noises are quite different from those of pure white noise. Therefore, these existing threshold determination methods are not suitable or adaptable for every noisy condition. Wahiba et al. [13] applied the wavelet threshold function directly to EMD denoising methods. The realisation of threshold-based denoising methods is achieved mainly through the hard threshold function, soft threshold function or interval threshold function. The hard threshold method does not address discontinuity in denoised IMFs. The soft threshold method solves discontinuity, but it lowers the peak, producing a peak clipping phenomenon. These methods produce signal distortion, which can affect their denoising performance.

In this paper, an integrated EMD adaptive threshold denoising method (IEMD-ATD) is proposed for the reduction of noise in ECGs. The main contributions are as follows: 1) An integrated EMD (IEMD) for decomposing ECG signals is proposed. The method utilises the CEEMDAN framework. Positive and negative noise pairs in a Gaussian distribution are utilised as assisting noises in the first stage. The noises remaining in the residual at each decomposition stage are used as the improved assisting noises for the corresponding stages. The proposed IEMD is able to suppress residual noise remaining in intrinsic mode functions (IMFs) and to reconstruct ECG signals accurately. The improved assisting noises eliminate spurious modes appearing in CEEMDAN and streamline the noise decomposition process compared with CEEMDAN. 2) To achieve precise and reasonable denoising, the IMFs decomposed by IEMD are divided into three groups: high-frequency noise predominant modes, useful information predominant modes and low-frequency motion artefact and baseline wandering predominant modes. High-frequency noise in the ECG signal is usually decomposed into lower-order IMFs, and the noise energy decreases as the IMF order number increases [20]. Therefore, the ratio of the noise energy in the IMF to the energy of the corresponding IMF is used to divide noisy IMFs (high-frequency noise predominant) and noise-free IMFs (useful information predominant). Motion artefacts and baseline wanderings in ECGs are usually distributed in higher-order IMFs and in the residual term. Based on the cyclostationarity of the ECG signals, the IMFs can be considered to contain no ECG components and can be discarded if their eigenperiod is larger than the RR interval of the ECG signal. 3) For the denoising of high-frequency noise predominant IMFs, an adaptive threshold determination method and a peak filtering denoising method are proposed. The adaptive threshold determination method is based on the principle of removing gross error by the Pauta criterion in the theory of error analysis. The threshold is calculated by removing the non-noise data in the IMF that are larger than 3σ of the corresponding IMF. Here, σ is the standard deviation. A new 3σ is calculated, and removing procedures are repeated until there are no data to be removed. Thus, the 3σ in the final loop is defined as the threshold that is used further to differentiate the noise and the useful values in the IMFs. The proposed peak filtering denoising method does not need a specific threshold function. In this method, the IMF to be denoised is bounded by the adaptive 3σ threshold. Peaks that are less than the threshold are set to zero. After the zero operation, new smaller peaks are formed. The process of setting peaks to zero is repeated until there is no peak less than the threshold. In addition to eliminating noise that is smaller than the threshold, the peak filtering method can also preserve the useful data that belong to useful peaks but are smaller than the threshold, which retains the detailed information in the QRS complex.

Synthetic and real ECG signals are used in experiments to test the performance of the proposed IEMD-ATD compared with the hard and soft threshold denoising methods based on EMD. The results indicate that the proposed method is more effective in improving the SNR and offers larger correlation coefficient, especially in effectively retaining the complete information of the QRS complex.

The rest of this paper is organised as follows. In Section 2, our proposed methods are described in detail. Then, the existing threshold denoising methods that are used for contrast experiments in this paper are described. The evaluation parameters utilised in our experiments are briefly introduced. In Section 3, the ECG data and noise used in the experiments are described. In Section 4, the results for both synthetic and real ECG signals and a discussion are offered to demonstrate the performance of the proposed denoising methods. Finally, conclusions are drawn in Section 5.

## 2. Methods

### 2.1 Integrated EMD adaptive threshold denoising method

**Fig 1:**
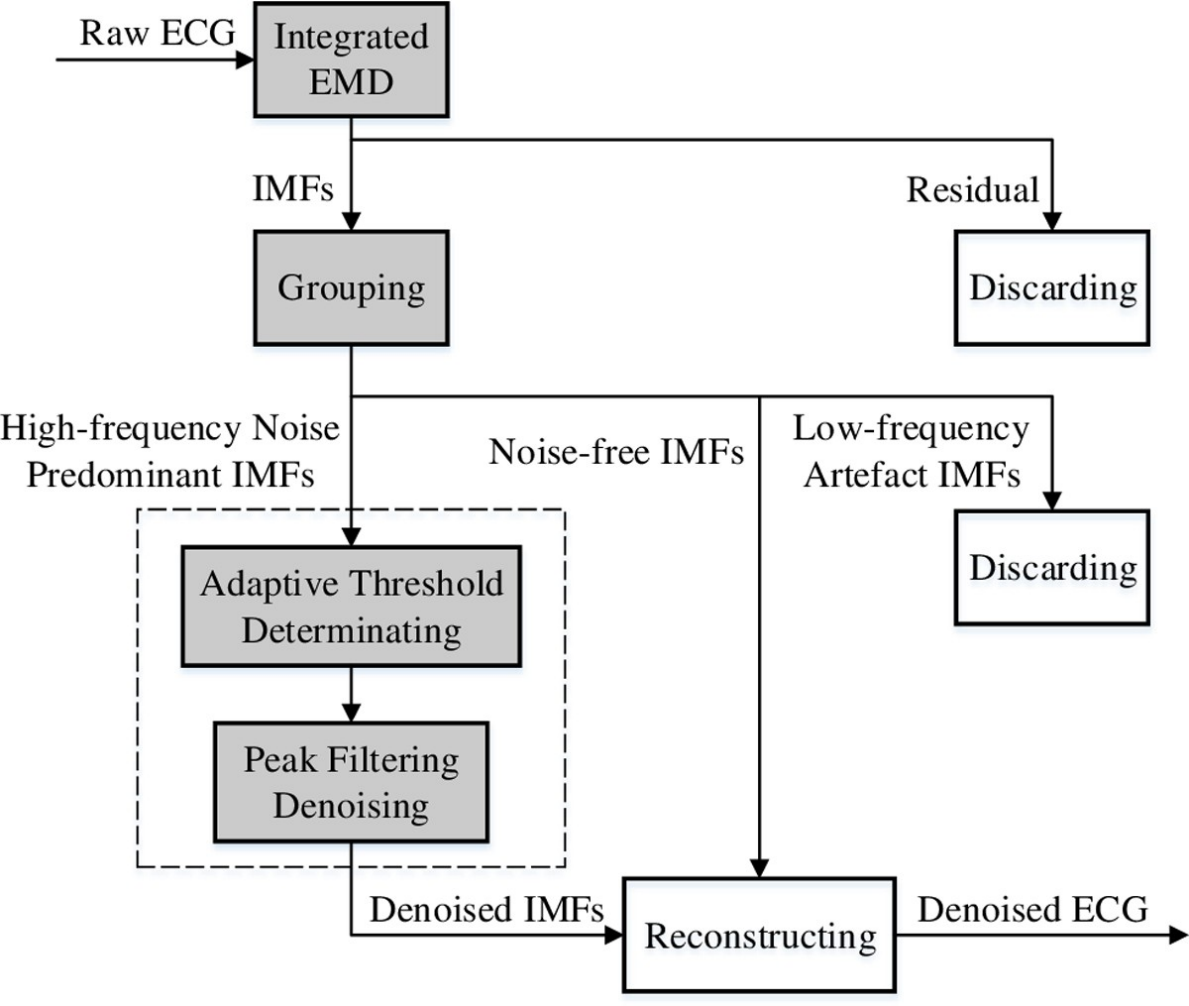
Flow of IEMD-ATD for ECG denoising.

In this section, an integrated EMD adaptive threshold denoising method (IEMD-ATD) is proposed, which is suitable for the reduction of noise in ECGs. IEMD-ATD contains four steps. First, the ECG is decomposed through integrated EMD (IEMD) into a set of IMFs and one residual term. Second, all of the IMFs are divided into three groups: high-frequency noise predominant IMFs, noise-free IMFs and IMFs with low frequency artefacts. Third, high-frequency noise predominant IMFs are denoised by the proposed peak filtering denoising method after the adaptive threshold is calculated. Finally, the denoised ECG signal is reconstructed by summing the denoised IMFs and the noise-free IMFs and directly discarding the IMFs with low-frequency artefacts. A flowchart of IEMD-ATD for ECGs is depicted in **Fig 1**. The boxes with grey backgrounds are the methods proposed in this paper.

#### 2.1.1 Integrated EMD

The integrated EMD (IEMD) proposed in this section utilises the framework of CEEMDAN and combines the CEEMD traits. First, IEMD uses complementary *N*(0,1) white Gaussian signals as the original assisting noises to suppress the residual noise in each IMF. The noises remaining in the residual in each decomposition stage are used as the improved assisting noises for the corresponding stages. Compared with CEEMDAN, IEMD avoids decomposing procedures of original assisting noises and enhances the computational efficiency. Additionally, the problem of spurious modes in CEEMDAN is solved. The IEMD procedure is depicted below:

1) Generate complementary *N*(0,1) white Gaussian noise, i.e., generate a random noise *W*(*n*) and then calculate its opposite number −*W*(*n*) as the other part of the assisting noise:

$$n(n)=\left[\begin{array}{c} 1\\ -1 \end{array}\right]w(n)=\left[\begin{array}{c} w(n)\\ -w(n) \end{array}\right]$$ (1)

where *n*(*n*) is the assisting noise. Because the assisting noises are generated in pairs, their number is always even. We define the relationship between the index of assisting noises and the white Gaussian noise as:

$$\begin{array}{cccc} n^{1}(n)=w_{1}(n) & n^{2}(n)=-w_{1}(n) & n^{3}(n)=w_{2}(n) & n^{4}(n)=-w_{2}(n) \end{array}\begin{array}{cc} & \cdots \end{array}$$ (2)

2) Given the ratio of the assisting noises *ε*, mix the original signal *x*(*n*) with the assisting noises generated in Step 1 to obtain the complementary ensemble signals:

$$x^{i}(n)=x(n)+\varepsilon n^{i}(n)\begin{array}{cc} & i=1\cdots I \end{array}$$ (3)

where *I* is the total number of ensemble signals.

3) Decompose *x*^*i*^(*n*) by EMD into $IMF_{1}^{i}(n)$ and calculate the average of $IMF_{1}^{i}(n)$ to obtain the 1st IMF of the original signal. *E*_1_(·) is an operator that refers to the 1st IMF extracted through EMD:

$$\hat{IMF_{1}}(n)=\frac{1}{I}\sum_{i=1}^{I}IMF_{1}^{i}(n)=\frac{1}{I}\sum_{i=1}^{I}E_{1}\left(x^{i}(n)\right)$$ (4)

4) Eliminate $\hat{IMF_{1}}(n)$ from *x*(*n*) to obtain the first residual:

$$r_{1}(n)=x(n)-\hat{IMF_{1}}(n)$$ (5)

5) Calculate the *i* ensemble residuals:

$$r_{1}^{i}(n)=x^{i}(n)-E_{1}\left(x^{i}(n)\right)$$ (6)

6) Average the *i* ensemble residuals:

$$\bar{r_{1}}(n)=\frac{1}{I}\sum_{i=1}^{I}r_{1}^{i}(n)$$ (7)

7) Subtract the average of the ensemble residuals from the ensemble residuals to obtain the noise remaining in each ensemble residual:

$$△r_{1}^{i}(n)=r_{1}^{i}(n)-\bar{r_{1}}(n)$$ (8)

8) Generate new ensemble signals using the residual in Step 4 and the noise signals in Step 7. Calculate the 2nd IMF of *x*(*n*):

$$\hat{IMF_{2}}(n)=\frac{1}{I}\sum_{i=1}^{I}E_{1}\left(r_{1}(n)+△r_{1}^{i}(n)\right)$$ (9)

9) The iteration formulas are given below:

$$r_{k}(n)=r_{k-1}(n)-\hat{IMF_{k}}(n)$$ (10)

$$r_{k}^{i}(n)=\left(r_{k-1}(n)+△r_{k-1}^{i}(n)\right)-E_{1}\left(r_{k-1}(n)+△r_{k-1}^{i}(n)\right)$$ (11)

$$\bar{r_{k}}(n)=\frac{1}{I}\sum_{i=1}^{I}r_{k}^{i}(n)$$ (12)

$$△r_{k}^{i}(n)=r_{k}^{i}(n)-\bar{r_{k}}(n)$$ (13)

$$\hat{IMF_{k}}(n)=\frac{1}{I}\sum_{i=1}^{I}E_{1}\left(r_{k-1}(n)+△r_{k-1}^{i}(n)\right)$$ (14)

Repeat Step 9 until there is at most one extremum in the residual *r*_*k*_(*n*) when it is no longer feasible to decompose. The final decomposition is depicted as follows:

$$x(n)=\sum_{k=1}^{K}\hat{IMF_{k}}(n)+r(n)$$ (15)

When using the noise remaining in the residual, there is no need to consider the ratio of the assisting noise before adding it into the residual to obtain the ensemble signal of the next step (see Formulas (9) and (14)).

The proposed integrated EMD is able to suppress the residual noise introduced in all of the IMFs due to the merit of complementary original assisting noises. By using the CEEMDAN framework, the integrated EMD retains the advantage of exact reconstruction. Moreover, we make use of the noise remaining in the residuals of each decomposition stage as subsequent assisting noises. As a result, a large number of procedures for decomposing subsequent assisting noises are eliminated compared with CEEMDAN, and the phenomenon of spurious modes due to the extra decomposition of white Gaussian noises is eliminated.

**Fig 2:**
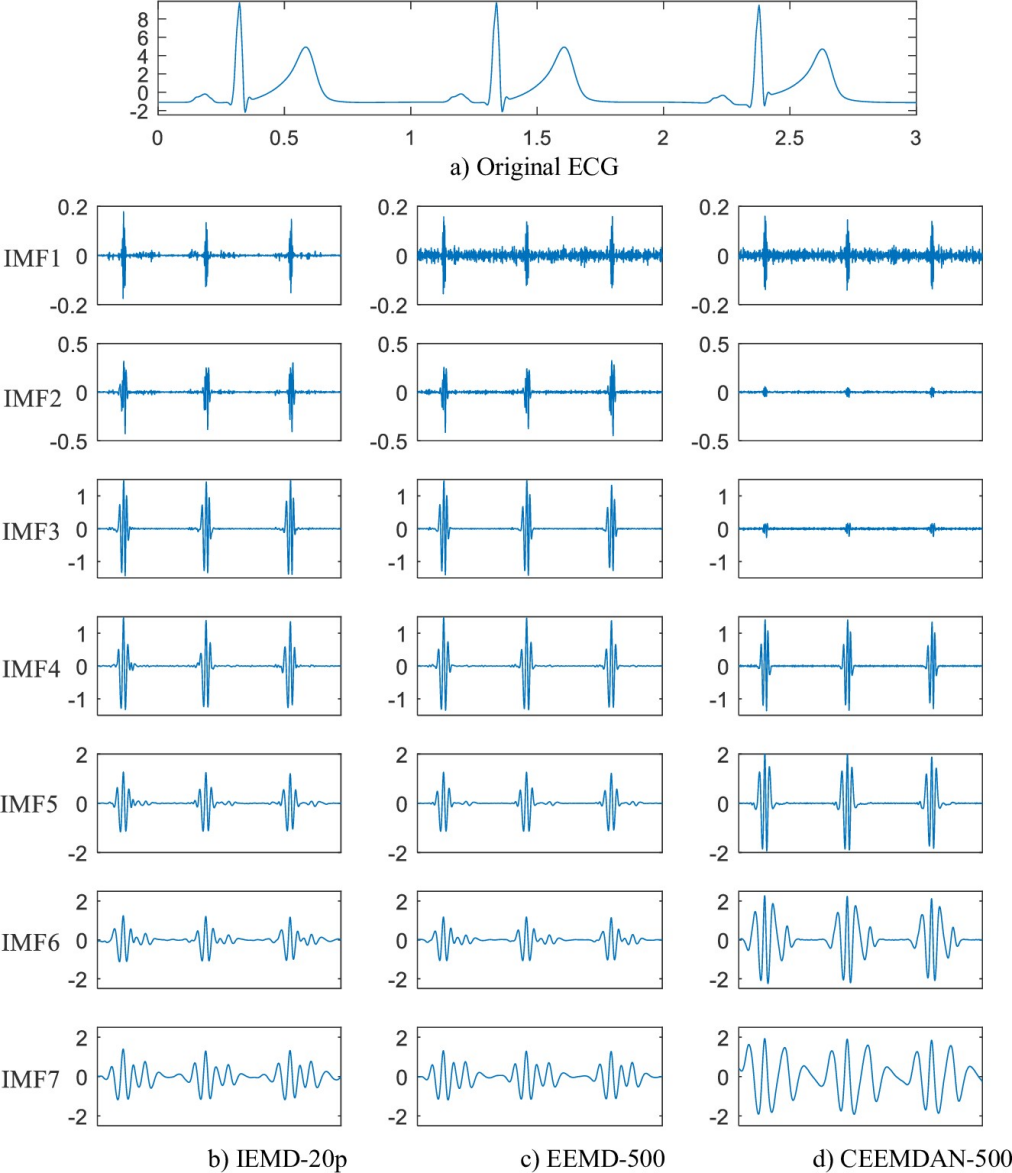
Comparison of the decomposition waveform (IMF1-IMF7).

**Fig 3:**
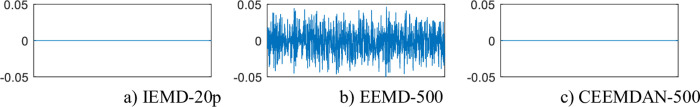
Waveforms of reconstruction errors.

**Fig 2** shows the waveforms of the first seven IMFs decomposed from an ECG signal, which is depicted in a). The decomposition methods are IEMD with 20 pairs of auxiliary noises (IEMD-20p in b)), EEMD with 500 auxiliary noises (EEMD-500 in c)) and CEEMDAN with 500 auxiliary noises (CEEMDAN-500 in d)). For IEMD, the noise remaining in the first IMF is the least, although the number of auxiliary noises is only 40. For CEEMDAN, IMF2 and IMF3 have similar characteristics to IMF1, which means that CEEMDAN produces spurious modes. **Fig 3** shows the reconstruction error. The errors are calculated by summing all of the IMFs from the three decomposition methods. The results show that only EEMD has numerous reconstruction errors, while IEMD and CEEMDAN have negligible reconstruction errors.

The decomposition times are displayed in **Table 1**, which shows the relative time of decomposition, which is the ratio of the computation time of the existing methods to the proposed IEMD-20p computation time. For example, taking the time of IEMD-20p as the standard time, EEMD-500 is 12.2 times slower than IEMD-20p. Generally, more auxiliary noise will lead to more time consumption. The computational circumstances are the same for all of the methods in the experiments. From **Figs 2 and 3 and Table 1**, it can be seen that IEMD not only saves decomposition time but also guarantees decomposition quality.

**Table 1:** Relative decomposition time compared with IEMD-20p.

Decomposition Method	Amount of Auxiliary Noise					
	40	100	200	300	400	500
EEMD	0.98	2.51	4.95	7.33	9.69	12.20
CEEMDAN	1.41	3.13	6.35	9.35	12.42	15.45

#### 2.1.2 IMF grouping

According to [19], the first several IMFs in an ECG consist of high-frequency components of the ECG contaminated with the components of high-frequency noise, while low-frequency noise, such as baseline wandering, mainly exists in the last few IMFs and in the residual term. The remaining IMFs have relatively little noise and contain useful information. Therefore, it is necessary to group the IMFs of the ECG into noisy IMFs, “pure” IMFs and low-frequency IMFs to process each group of IMFs differently. In this section, a new IMF grouping method is proposed. Two IMF boundaries will be calculated through this method.

*a) IMF boundaries with high-frequency noise*. As previously mentioned, high-frequency noise in the ECG signal is usually decomposed into lower-order IMFs, and the noise energy decreases as the order number of the IMF increases. Therefore, the boundary of high-frequency noise predominant IMFs can be determined by finding the IMF with the least proportion of the noise in the IMF. On the one hand, the proposed method estimates the proportion of the noise in the IMF by calculating the ratio of the standard deviation of the IMF to that of the noise. When the ratio reaches the maximum, the noise in the IMF is the least. On the other hand, the difference between the standard deviation of the IMF and that of the noise reaching the maximum also reflects the minimum proportion of the noise in the IMF. When the ratio and the difference reach the maximum, two corresponding order numbers of the IMF (i.e., *k*) are obtained. The proposed method determines the boundary of high-frequency noise predominant IMFs by rounding up the mean of the two order numbers to the nearest integer. The details of the method are described as follows:

1) Calculate the standard deviation of an IMF

$$\sigma_{k}=\sqrt{\frac{1}{N-1}{\sum_{n=1}^{N}\left(IMF_{k}(n)-\bar{IMF_{k}(n)}\right)}^{2}}$$ (16)

where *k* represents the order number of IMFs. *N* is defined as the data length of the IMF.

2) Estimate the standard deviation of noise in the corresponding IMF

$${\hat{\sigma }}_{k}=\frac{median\left(\left|IMF_{k}(n)-\bar{IMF_{k}(n)}\right|\right)}{0.6745}$$ (17)

3) Estimate the boundary of noise dominating IMF

$$K_{B}=\left\lceil \frac{\underset{k}{\arg\max}\left(\sigma_{k}/{\hat{\sigma }}_{k}\right)+\underset{k}{\arg\max}\left(\sigma_{k}-{\hat{\sigma }}_{k}\right)}{2}\right\rceil$$ (18)

The IMFs with orders smaller and equal to *K*_*B*_ are the high-frequency noise predominant IMFs.

*b) IMF boundaries with low-frequency baseline wandering*. An ECG is a quasi-periodic signal. The characteristics of ECGs, such as PQRST waves, repeat with time. Therefore, IMFs with components with oscillation frequencies lower than the ECG repeating frequency should be considered low-frequency noise, such as baseline wandering. In this paper, we identify the IMFs belonging to low-frequency noises by comparing the average period of R peaks (the average of RR intervals) in the original ECG with the average period of the IMFs. The definition of the average period of R peaks in ECG is:

$$\bar{T_{R}}=\frac{N}{N_{R}}$$ (19)

where *N*_*R*_ is the number of R peaks in the original ECG. *N* is the data length of the original ECG.

Similarly, the average period of the IMF is defined as:

$$\bar{T_{IMF}}=\frac{N}{N_{\max}}$$ (20)

where *N*_*max*_ represents the number of local maxima for an IMF.

Hence, the boundary of low-frequency baseline wandering is the first-order number of the IMF whose average period satisfies $\bar{T_{IMF}}>\bar{T_{R}}$. Baseline wandering is eliminated by discarding all of the IMFs with $\bar{T_{IMF}}>\bar{T_{R}}$ and the residual term.

#### 2.1.3 Adaptive threshold determination based on the 3σ criterion

In EMD threshold denoising methods, useful information is generally considered to be contained in larger values of noisy IMFs, while noise is contained in smaller values. Threshold denoising methods need to determine a threshold between the useful information and the noise in the IMF, which should be done according to the corresponding categories of noise. In this section, an adaptive threshold determination method based on the 3σ criterion (Pauta criterion) is proposed. The method temporarily removes the non-noisy useful values in the IMFs that are larger than 3σ. The remaining noisy values are used to calculate the new 3σ. By repeating the process until no value needs to be removed, the final 3σ of the “pure” noise is the threshold calculated adaptively for distinguishing noise and useful values. The complete procedure of the adaptive threshold determination method is as follows:

1) Calculate the standard deviation of the IMF:

$$\sigma_{0}=\sqrt{\frac{1}{N-1}\sum_{n=1}^{N}{\left(IMF(n)-\bar{IMF(n)}\right)}^{2}}$$ (21)

2) Remove the values that are larger than 3***σ***0 in $IMF(n)-\bar{IMF(n)}$. The remaining values are regarded as the new noisy signal *IMF*^1^(*n*). The length is represented as *N*_1_, which is the number of remaining values.

3) Calculate the standard deviation of *IMF*^1^(*n*):

$$\sigma_{1}=\sqrt{\frac{1}{N_{1}-1}\sum_{n=1}^{N_{1}}{\left(IMF^{1}(n)-\bar{IMF^{1}(n)}\right)}^{2}}$$ (22)

4) Generally, the values in $IMF^{i-1}(n)-\bar{IMF^{i-1}(n)}$ that are larger than 3***σ***_***i***−1_ are removed. The remaining values are regarded as the new noisy signal *IMF*^*i*^(*n*) of which the length is *N*_*i*_, which is the number of remaining values. The standard deviation of *IMF*^*i*^(*n*) is:

$$\sigma_{i}=\sqrt{\frac{1}{N_{i}-1}\sum_{n=1}^{N_{i}}{\left(IMF^{i}(n)-\bar{IMF^{i}(n)}\right)}^{2}}$$ (23)

Repeat Step 4) until there is no value in $IMF^{i}(n)-\bar{IMF^{i}(n)}$ larger than 3***σ***_*i*_. At this moment, 3***σ***_*i*_ is the final threshold determined:

$$\lambda =3\sigma_{i}$$ (24)

By repeating the iteration, the 3*σ* calculated in each step will gradually decrease. Much “purer noise” will be screened out by each repeat. Therefore, the last 3***σ***_*i*_ represents the final threshold.

It should be stressed that *N*_*i*_ in Step 4 gradually decreases as *i* increases. This is because the removed values no longer participate in the subsequent calculations of 3σ.

#### 2.1.4 Peak filtering denoising method

The hard and soft threshold denoising methods both consider values smaller than the threshold as noise, which should be set to zero. However, for ECG signals, every value belonging to useful waves should be retained, even if some of them are smaller than the threshold. Discarding these values leads to the loss of local information of the ECG waveform as well as discontinuity. To retain the values containing useful information but smaller than the threshold, a peak filtering denoising method is proposed. The proposed method is described as follows:

1. Calculate all of the local maxima and local minima of an IMF;
2. Compare the absolute values of the local maxima and minima with the adaptive threshold *λ* determined in the previous section, separately. Set the extrema whose absolute values are smaller than the threshold to zero and obtain a half-processed IMF;
3. Calculate all of the local maxima and minima of the half-processed IMF obtained in Step 2;
4. Compare the absolute values of the local maxima and minima in Step 3 with the same threshold *λ*, set the extrema whose absolute values are smaller than the threshold to zero and obtain a further half-processed IMF.

Repeat Steps 3) and 4) until there are no more local maxima and minima to be set to zero. The remaining IMF is the denoised IMF.

By repeatedly filtering the peaks (local maxima and local minima) that are smaller than the threshold, the proposed method can retain the full QRS information and reduce noise in the ECG signal.

**Fig 4:**
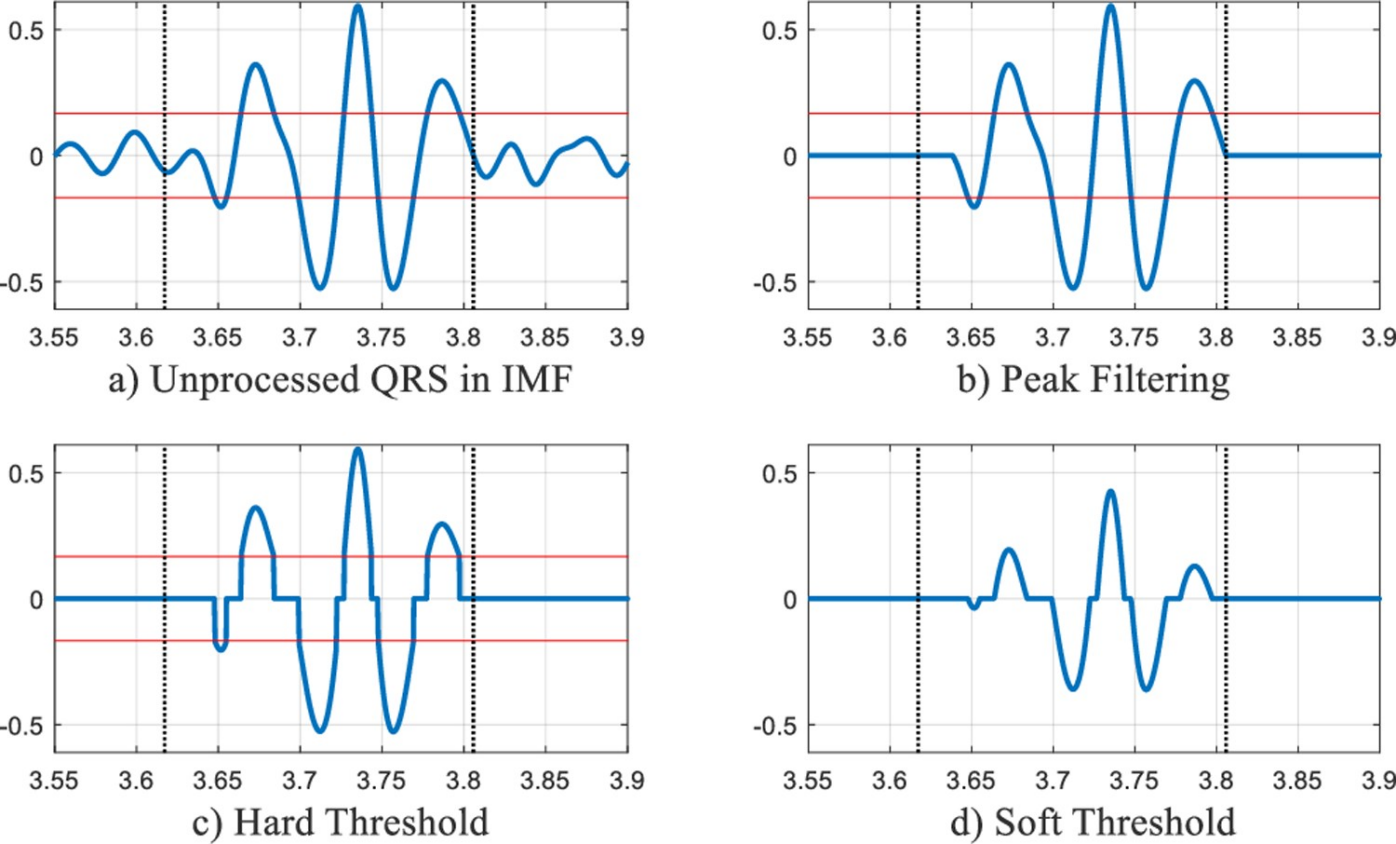
Comparison of QRS detail waveforms denoised by three denoising methods.

**Fig 4** displays detailed QRS waveforms in an IMF. The first of these signals is an unprocessed QRS waveform, and the other three are QRS waveforms processed by three denoising methods. The two horizontal lines in **Fig 4** are drawn according to the threshold. The zone determined by the two vertical dotted lines contains useful information of the QRS complex in the IMF. Therefore, within the zone, any value smaller than the threshold should still be retained.

It can be clearly seen that for the hard threshold denoising method, there are discontinuities; for the soft threshold denoising method, the phenomenon of reducing the amplitude appears. For the proposed peak filtering denoising method, the problems caused by hard and soft threshold denoising methods are solved effectively, and the full QRS information is retained.

### 2.2 Comparison of EMD threshold denoising methods

In this section, we briefly introduce the EMD threshold denoising methods, which are compared with the proposed IEMD-ATD. Universal threshold [23] and EMD customised threshold [13] are used in the EMD threshold denoising methods. The universal threshold is a general method and is introduced directly from wavelet threshold denoising methods, while the EMD customised threshold is developed specifically for EMD threshold denoising, which is only suitable for EMD. Hard and soft threshold methods are utilised as denoising strategies. Although there are many interval threshold methods, some of the parameters have to be amended according to the specific circumstances. Only the hard and soft threshold denoising methods are used widely and practically, so we use hard and soft threshold denoising methods as the general comparison methods.

The formula of the universal threshold in EMD is:

$$\lambda_{i}={\hat{\sigma }}_{i}\sqrt{2\ln(N)}$$ (25)

where *N* is defined as the number of signal samples. ${\hat{\sigma }}_{i}$ is the estimation of the square root of the noise energy in the *i*-th IMF, which is estimated by:

$${\hat{\sigma }}_{i}=\frac{median\left(\left|IMF_{i}(n)-\bar{IMF_{i}(n)}\right|\right)}{0.6745}$$ (26)

$$\bar{IMF_{i}(n)}=\frac{1}{N}\sum_{n=1}^{N}IMF_{i}(n)$$ (27)

Each ${\hat{\sigma }}_{i}$ is estimated from the corresponding IMF.

EMD customised thresholds are proposed in reference [13]. The formula is described as follows:

$$\lambda_{i}=C\sqrt{E_{i}2\ln(N)}$$ (28)

where *C* is a constant determined according to the real situation. As recommended in [13], *C* is normally set to 0.5. *N* represents the length of the signal. ***E***_***i***_ is the estimation of the noise energy in the *i*-th order of IMF:

$$E_{i}=\frac{E_{1}}{0.719}2.01^{-i}\begin{array}{cc} & i=2,3,4\cdots K \end{array}$$ (29)

where ***E***_1_ is the noise energy estimation in IMF_1_ calculated by:

$$E_{1}={\left(\frac{median\left(\left|IMF_{1}(n)-\bar{IMF_{1}(n)}\right|\right)}{0.6745}\right)}^{2}$$ (30)

$$\bar{IMF_{1}(n)}=\frac{1}{N}\sum_{n=1}^{N}IMF_{1}(n)$$ (31)

The hard threshold denoising method directly sets the values less than the threshold *λ* to zero and keeps the other values. The formula is as follows:

$$\hat{x}(n)=\{\begin{array}{c} x(n)\\ 0 \end{array}\begin{array}{cc} & \begin{array}{c} \left|x(n)\right|\geq \lambda \\ \left|x(n)\right|<\lambda \end{array} \end{array}$$ (32)

The soft threshold denoising method [24] smoothens the denoised signal. The formula is described below:

$$\hat{x}(n)=\{\begin{array}{c} sign\left[x(n)\right]\left(\left|x(n)\right|-\lambda \right)\\ 0 \end{array}\begin{array}{cc} & \begin{array}{c} \left|x(n)\right|\geq \lambda \\ \left|x(n)\right|<\lambda \end{array} \end{array}$$ (33)

where *sign* denotes the sign function, which is defined as:

$$sign(x)=\{\begin{array}{c} 1\\ -1 \end{array}\begin{array}{cc} & \begin{array}{c} x\geq 0\\ x<0 \end{array} \end{array}$$ (34)

### 2.3 Evaluation parameters

To evaluate the performance of our methodology quantitatively, three evaluation parameters are utilised in our paper to assess both the proposed IEMD-ATD and the EMD threshold denoising methods used for comparison.

The signal-to-noise ratio (SNR) represents the ratio of useful signal energy to noise energy. The definition of SNR is:

$$SNR=10\text{lg}\left(\frac{\sum_{n=1}^{N}s^{2}(n)}{\sum_{n=1}^{N}n^{2}(n)}\right)$$ (35)

where *s*(*n*) is the original noise-free signal and *n*(*n*) is the noise mixed in the signal. Their relationships are as follows:

x(n)=s(n)+n(n) (36)

where *x*(*n*) is the noisy signal.

By inspecting the improvement of the SNR, we evaluate the performance of the denoising algorithms. The SNR improvement is defined as [25]:

$$SNR_{ipv}=SNR_{denoised}-SNR_{orignal}$$ (37)

SNR improvement is a parameter that evaluates performance from the aspect of energy. There is also a need to evaluate waveform shape. The correlation coefficient (*C*_*R*_) is chosen as another indicator in our experiments. A higher *C*_*R*_ indicates a higher similarity of delineation. The definition of *C*_*R*_ is:

$$C_{R}=\frac{\sum_{n=1}^{N}\left(x_{d}(n)-\bar{x_{d}(n)}\right)\left(s(n)-\bar{s(n)}\right)}{\sqrt{\sum_{n=1}^{N}{\left(x_{d}(n)-\bar{x_{d}(n)}\right)}^{2}}\sqrt{\sum_{n=1}^{N}{\left(s(n)-\bar{s(n)}\right)}^{2}}}$$ (38)

where *x*_*d*_(*n*) denotes the denoised signal and $\bar{x_{d}(n)}$ and $\bar{s(n)}$ represent the average of the corresponding signal.

Relative differences of signal energy (RDE) is a parameter used to evaluate the similarity of amplitude indirectly. It is an auxiliary parameter because it needs to combine the results of the correlation coefficient. RDE is defined as:

RDE=∑n=1Nxd2(n)−∑n=1Ns2(n)∑n=1Ns(n)2 (39)

RDE reflects the relationship between the energy of the denoised signal and that of the original signal. When the correlation coefficient is not very small, a lower RDE implies a higher similarity of amplitude.

## 3. Materials

### 3.1 Source of ECG data

#### a) Synthetic data

**Fig 5:**
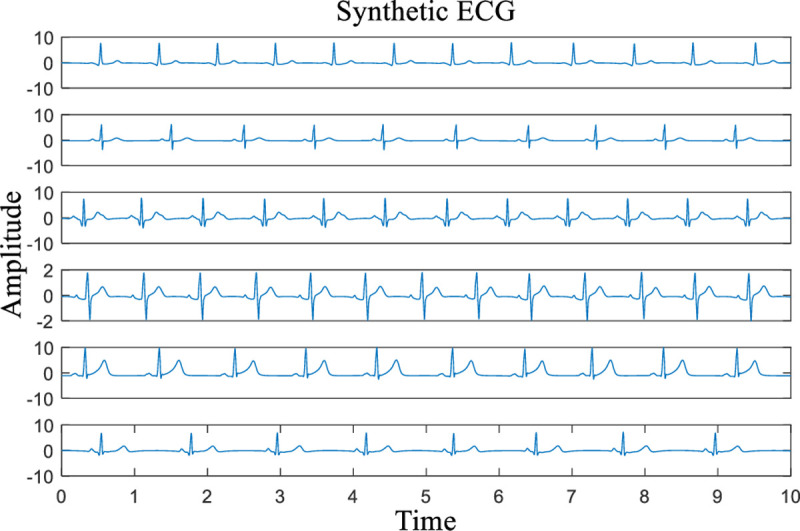
Synthetic ECG signals with a sampling rate of 360 Hz in 10 s (from top to bottom, the names of the signals are Syn00, Syn01, Syn02, Syn03, Syn04 and Syn05).

FECGSYN is part of the Open Source ECG Toolbox package (OSET). It is an updated version of ECGSYN and is built upon the work of McSharry et al. [26] and Sameni et al. [27]. Both adult ECGs and noninvasive foetal ECGs can be generated via FECGSYN. In this paper, 6 synthetic ECG signals with different characteristics are generated by FECGSYN for our subsequent experiments. The different characteristics include heart rate, shape of the QRS complex and relative amplitude of the T waves. The parameters corresponding to these characteristics are chosen randomly. The sampling rate is set to 360 Hz, and the sampling time is 10 seconds. The waveforms of the 6 synthetic ECG signals are depicted in **Fig 5** with the names Syn00, Syn01, Syn02, Syn03, Syn04 and Syn05 (from top to bottom).

#### b) Real data

The MIT-BIH Arrhythmia Database (MITDB) [28] collects various representative records sampled in clinical practice. In our research, 10 sets of records in MITDB are selected for our experiments. To the best extent possible, we choose different morphologies of ECG signals as experimental data, such as heart rate, shape of the QRS complex and relative amplitude of T waves. Moreover, to calculate SNR more accurately to verify the denoising effect in the following experiments, signals with as little noise as possible are chosen. The sampling rates of these records are all 360 Hz. We cut off and selected an interval of 10 seconds in each set of records, which are listed in **Table 2**. The data can be downloaded through [29].

**Table 2:** Real ECG data chosen from the MIT-BIH Arrhythmia Database.

Record Number	100	101	103	112	113	115	117	119	122	123
Starting Time	0:10	0:10	0:10	0:10	0:10	1:00	0:10	1:40	0:10	0:10
Channel Name	MLII	MLII	MLII	MLII	MLII	MLII	MLII	MLII	MLII	MLII

### 3.2 Source of noises

Noise introduction is inevitable when an ECG is recorded. The Noise Stress Test Database (NSTDB) [30, 31] in MIT-BIH contains three sets of noises (the names of the records are “bw”, “em”, and “ma”), and each of them has two signals recorded simultaneously. Record ‘bw’ contains predominant baseline wandering; record ‘em’ contains the artefacts from electrode motion (baseline wandering and muscle noise included as well); record ‘ma’ contains primarily muscle artefacts (mainly electromyogram noise).

**Fig 6:**
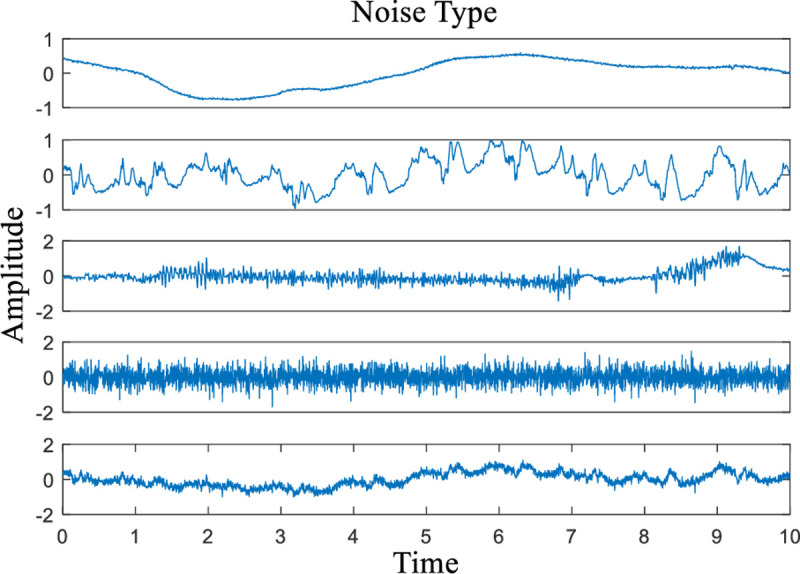
Five categories of noise used in the experiments (from top to bottom, the noise types are BW, ECN, EMG, WN and HN).

In this paper, the noises under study are baseline wandering (BW), electrode contact noise (ECN), electromyogram noise (EMG), white noise (WN) and hybrid noise (HN). Among them, BW, ECN and EMG are chosen from the “bw”, “em”, and “ma” in NSTDB, respectively. The sampling rates of the three kinds of noises are all 360 Hz. A 10 s interval of noise data from each of them is selected. WN is generated by the random function in MATLAB 2016. Finally, we generate HN by adding the four categories of noise. Each individual category of noise is considered separately in the subsequent denoising experiments, so for hybrid noise, we perform a simple treatment in which hybrid noise is generated by summing four kinds of noise directly. The waveforms of the five noises are depicted in **Fig 6**.

## 4. Results and discussion

### 4.1 Results of eliminating baseline wandering by IEMD-ATD

**Fig 7:**
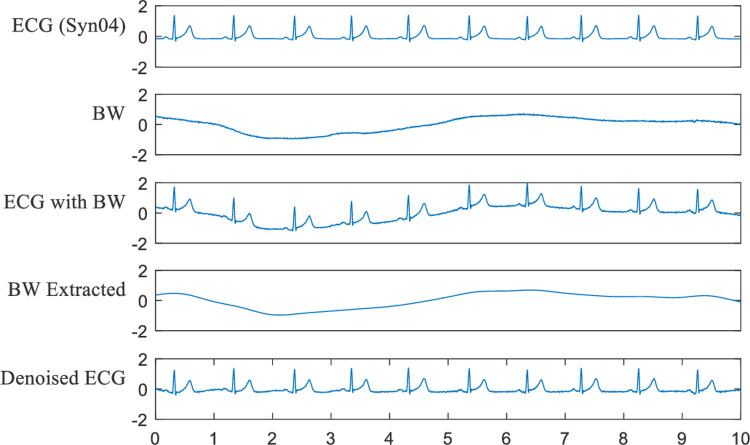
Effect of eliminating BW for Syn04 with BW at -5 dB.

In this section, baseline wandering elimination is inspected. The results of Syn04 mixed with BW at -5 dB are depicted in **Fig 7**. From the waveform of the denoised ECG, we can see that IEMD-ATD eliminates the baseline wandering successfully. The “BW Extracted” are almost the same as BW, which was originally introduced.

**Fig 8:**
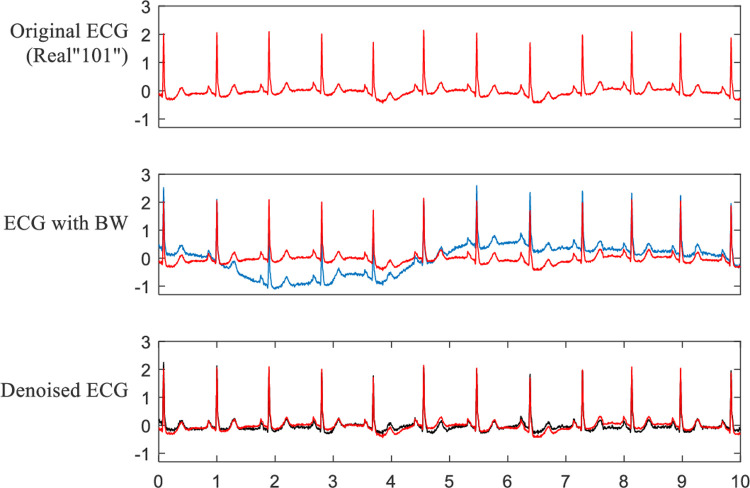
Effect of eliminating BW for real “101” with BW at -5 dB.

**Tables 3** and **4** display the SNR improvement by eliminating BW for synthetic and real ECG signals, respectively. The original SNR is -5 dB, -2 dB, 2 dB, 5 dB and 10 dB. From **Table 3**, it can be seen that the BW elimination results are extremely satisfactory. The lower the original SNR is, the higher the SNR improvement is. Except for Syn05 at 10 dB, all of the other experimental results have SNR improvements of more than 14 dB. As seen from **Table 4**, the SNR improvement results of the real ECG signals are also very good but not as good as those of the synthetic ECG signals. This is because the original real ECG signals are from the MITDB and contain some small noise, including baseline wandering, which is also eliminated effectively in the denoising process. As a result, if the original real ECG signal is used as the *clean* signal to calculate the SNR, it will reduce the SNR improvement. However, in fact, the denoised ECG in our method is closer to a *clean* ECG compared with the original real ECG. This is illustrated in **Fig 8**. The red lines represent the original real ECG. The blue line represents the ECG with BW, and the black line represents the denoised ECG by IEMD-ATD.

**Table 3:** SNR improvement from eliminating BW for synthetic ECGs.

ECG (Syn)	Original SNR				
	-5 dB	-2 dB	2 dB	5 dB	10 dB
00	21.13	20.07	18.42	17.85	15.33
01	21.44	21.90	21.21	18.14	19.32
02	19.97	21.88	19.54	18.60	17.28
03	20.49	20.92	18.57	16.97	14.74
04	19.09	20.12	19.03	18.54	10.03
05	21.03	19.98	20.18	16.15	9.64

**Table 4:** SNR improvement from eliminating BW for real ECGs.

ECG (Real)	Original SNR				
	-5 dB	-2 dB	2 dB	5 dB	10 dB
100	19.01	17.67	14.92	13.38	9.68
101	14.32	11.83	8.20	6.11	0.32
103	16.53	14.06	10.46	7.56	2.45
112	16.03	13.45	10.20	7.68	3.09
113	19.47	17.54	9.46	11.02	5.93
115	16.58	14.15	10.92	8.84	4.44
117	13.72	9.25	4.22	6.75	0.57
119	20.49	20.05	18.24	16.02	11.33
122	19.50	19.12	15.86	13.71	9.40
123	16.42	12.82	10.24	6.54	4.04

Both the figures and the tables indicate that IEMD-ATD is a powerful method for eliminating BW in ECG signals. We can conclude that BW elimination by IEMD-ATD is successful and effective.

Generally, IMFs without ECG components have no QRS complex characteristics. Additionally, P waves do not appear in higher-order IMFs. T waves can be very large in certain circumstances; they may exist in higher-order IMFs, but R peaks are predominant in amplitude. Thus, R peaks provide local maxima (or minima) in higher-order IMFs. Therefore, if the number of local maxima in an IMF is smaller than that of the R peaks in the ECG, the component in this IMF is not the relevant ECG information; the component should be some low-frequency noise. The average RR interval is one of the parameters that can reflect the number of R peaks. In our method, we use the signal data number instead of the signal time because it is more convenient for data processing.

### 4.2 Performance evaluation for eliminating high-frequency noise by IEMD-ATD

In this section, the ECGs in the experiments are both synthetic and real signals according to **Section 3.1**. The current existing denoising methods that are utilised to compare with our IEMD-ATD are as follows:

1. Hard universal thresholding (UT-H);
2. Soft universal thresholding (UT-S);
3. Hard EMD customised thresholding (Cust-H);
4. Soft EMD customised thresholding (Cust-S).

In the experiments, the decomposition procedures of the above methods are all implemented by EMD. Both the grouping of IMFs and the elimination of baseline wandering are implemented by the method proposed in this paper, which focuses on testing the denoising effect of IEMD-ATD on high-frequency noise.

The noise types used in the experiments are electrode contact noise (ECN), electromyogram noise (EMG), white noise (WN) and hybrid noise (HN), as identified in **Section 3.2**. Three measurements (SNR Improvement, Correlation Coefficient and Relative differences of signal Energy) described in **Section 2.3** are used to evaluate denoising performance.

#### 4.2.1 Results for synthetic data

It should be pointed out that the noises ECN, EMG and HN include both high-frequency and low-frequency components. This is because ECN and EMG derive from real databases. Section 4.2 offers the denoising results from eliminating both high-frequency and low-frequency noise components. Then, to investigate eliminating only high-frequency noise by our IEMD-ATD, the experiments neglect the existence of low-frequency noises and regard them as a part of the ECG to not eliminate them.

**Fig 9:**
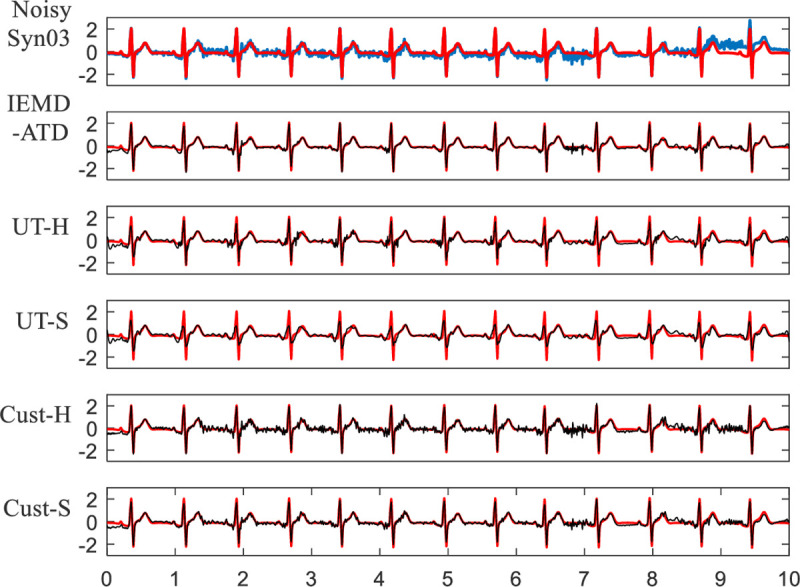
Waveforms of denoised ECGs for Syn03 with EMG noise at 5 dB in the 5 denoising methods.

**Fig 10:**
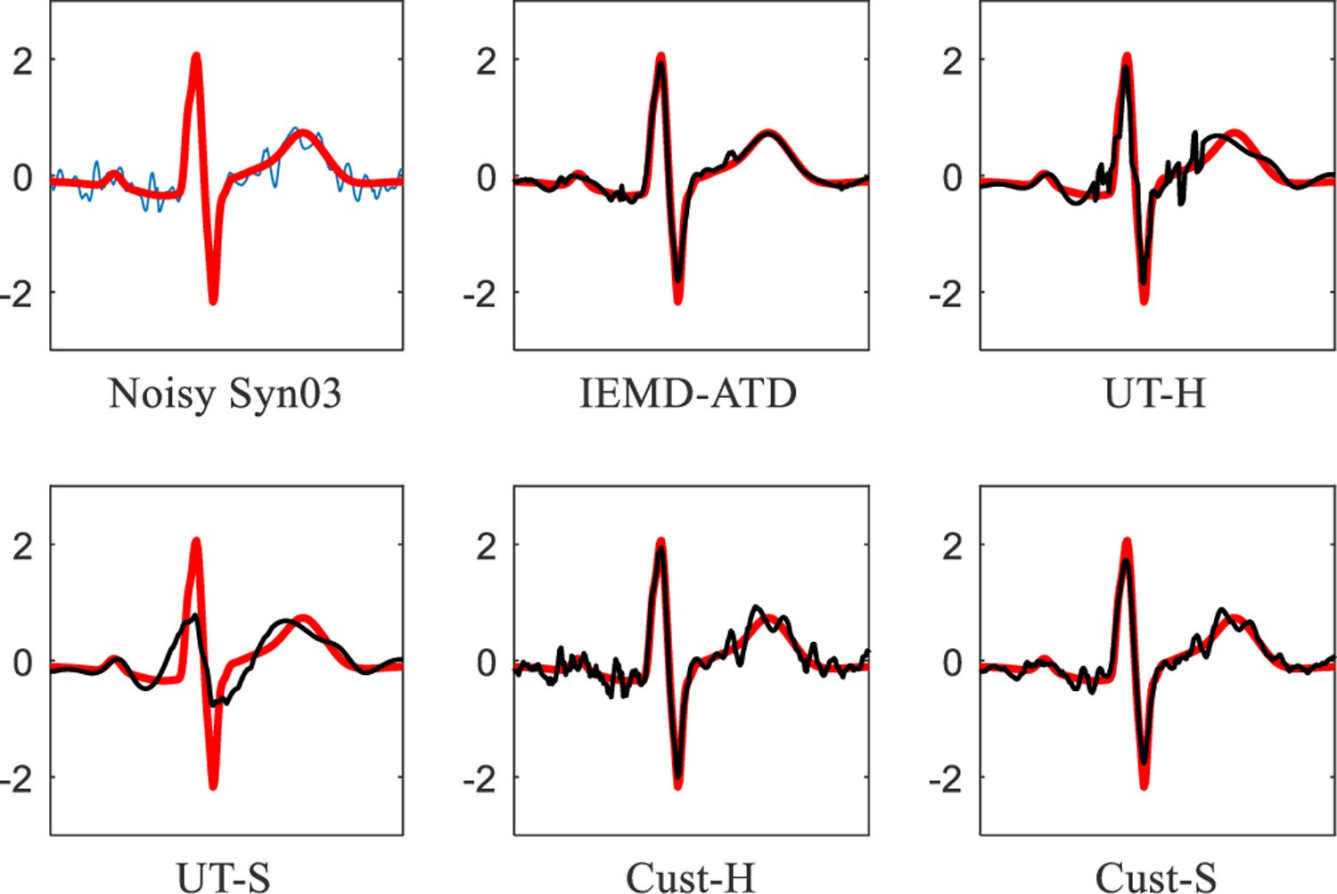
Details of waveforms between 2.4 and 3.05 seconds from Fig 9.

**Fig 11:**
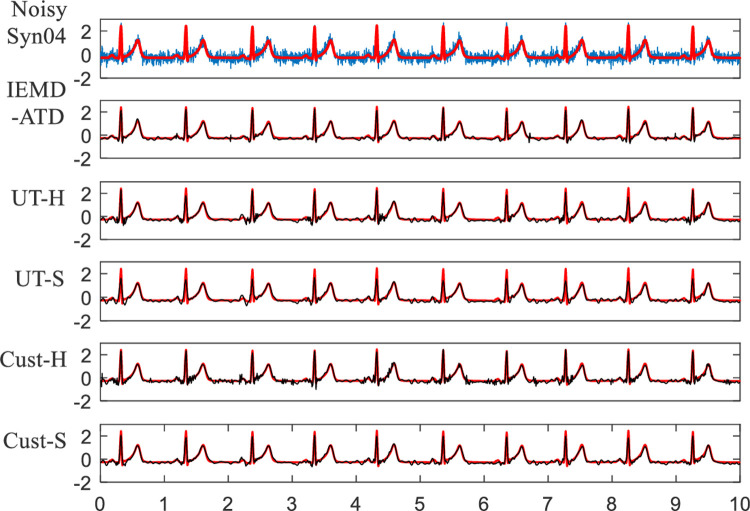
Waveforms of denoised ECG for Syn04 with WN at 5 dB in the 5 denoising methods.

**Fig 12:**
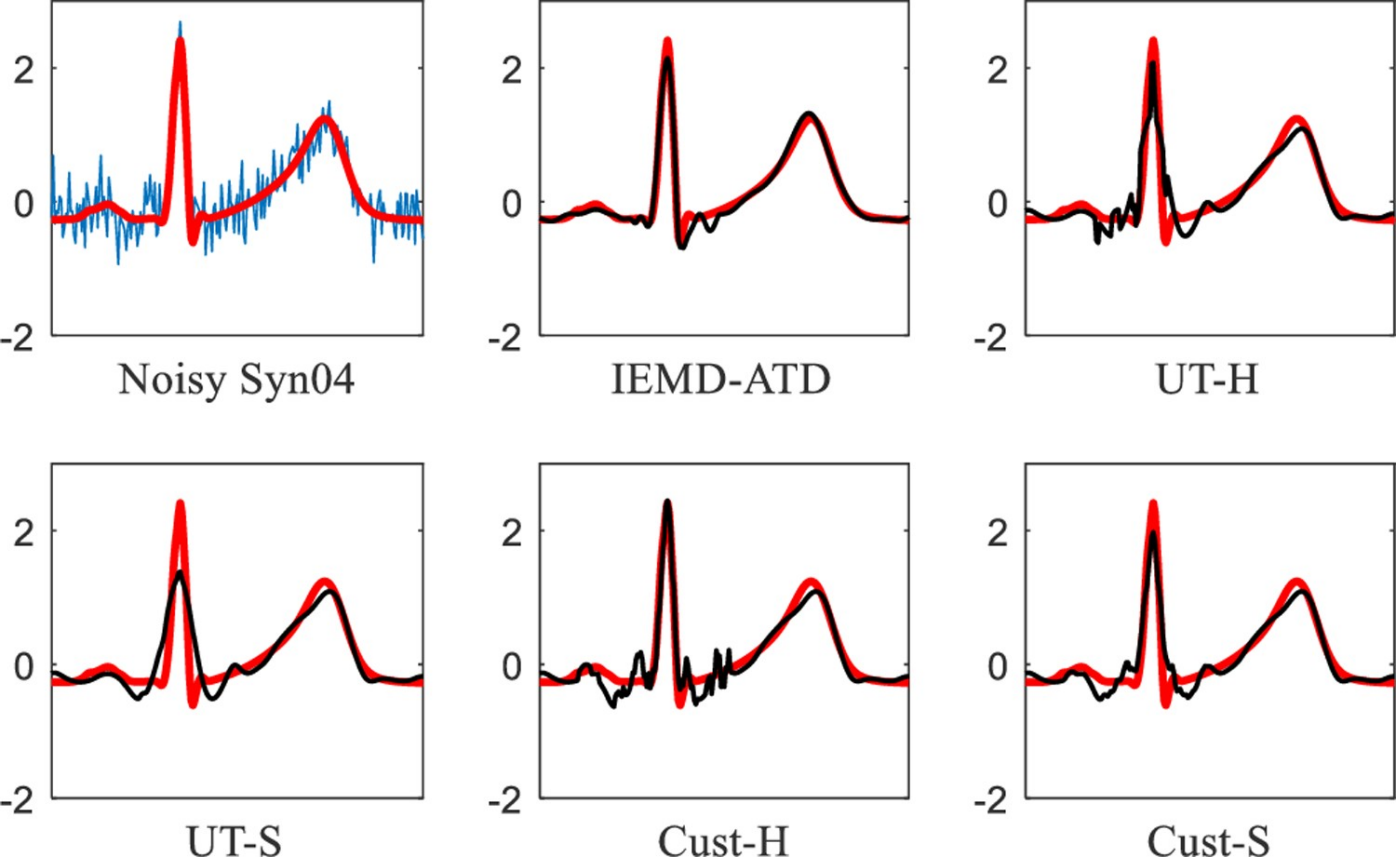
Details of waveforms between 7.05 and 7.7 seconds from Fig 11.

The waveforms of the denoised ECG are displayed from **Figs 9** to **12**. In **Figs 9** and **10**, Syn03 with 5 dB EMG noise is used. The red lines in each figure represent the original noise-free ECGs, while the blue line represents the noisy ECGs. The black lines represent the denoised ECGs by IEMD-ATD and the corresponding EMD denoising methods. In **Fig 9**, it can be seen that IEMD-ATD removes the most noise and retains the most signal information. **Fig 10** shows the local waveform details between 2.4 and 3.05 seconds. IEMD-ATD provides the best denoising performance with the cleanest noise filtering and the best coincidence degree between the QRS complex as well as the R peak in the denoised signal and those in the original clean signal. UT-S reduces the amplitude of the R and S peaks. UT-H and Cust-S show little loss of peak information, and considerable noise remains from the R peak to the T peak. For Cust-H, a large amount of noise still exists within the whole time range.

**Fig 11** shows the waveforms of denoised Syn04 with WN at 5 dB. The local waveform details of the denoised ECG for the five methods between 7.05 and 7.7 seconds are depicted in **Fig 12**. We can see that for UT-S and Cust-S, the amplitudes of the R peaks are obviously reduced. Considerable noise remains in Cust-H. For UT-H, the details around the Q and S waves are not good. The denoised ECG from the proposed IEMD-ATD has the highest similarity with the original noise-free ECG, which indicates that IEMD-ATD has the best denoising performance.

SNR improvements of the 5 denoising methods for 6 synthetic ECGs (Syn00-Syn05) are displayed in **Table 5**. The original SNR of the noisy ECG is 5 dB. The noises used here are EMG and WN. “Avg” in the table represents the average value of SNR improvement calculated by the 6 synthetic ECG signals. According to **Table 5**, it can be seen that the denoising performance of IEMD-ATD is the best with the highest SNR improvement and average for eliminating both EMG and WN.

**Table 5:** SNR improvement of 5 denoising methods for synthetic ECGs contaminated with EMG and WN at 5 dB.

Noise Type	ECG (Syn)	Denoising Method				
		IEMD -ATD	UT-H	UT-S	Cust-H	Cust-S
EMG	00	8.71	4.26	2.22	4.06	5.07
	01	7.55	5.64	3.24	4.91	6.05
	02	7.17	5.32	3.91	4.85	5.79
	03	8.33	1.85	-0.49	4.20	5.27
	04	8.10	6.42	4.59	5.06	6.94
	05	7.50	4.35	1.94	4.15	5.20
	Avg	7.89	4.64	2.57	4.54	5.72
WN	00	10.98	4.26	1.44	6.42	5.55
	01	9.43	2.94	0.21	5.12	3.62
	02	8.63	4.71	2.03	6.11	5.18
	03	8.56	4.16	1.36	5.51	5.06
	04	10.24	5.23	3.36	7.34	6.93
	05	9.94	5.70	2.59	6.65	5.95
	Avg	9.63	4.50	1.83	6.19	5.38

To verify the results of all the data mentioned in this paper, **Table 6** displays the average SNR improvement of the 6 synthetic ECGs (Syn00-Syn05) with the original SNRs of -5 dB, -2 dB, 2 dB, 5 dB and 10 dB. The numerical results indicate that IEMD-ATD has the best performance with the highest average SNR improvement. According to the table, the denoising effects for EMG and WN using our method are prominently better than those of the other methods.

**Table 6:** Average SNR improvement of 6 synthetic ECGs (Syn00-Syn05).

Original SNR	Noise Type	Denoising Method				
		IEMD -ATD	UT-H	UT-S	Cust-H	Cust-S
-5 dB	ECN	2.83	2.68	2.66	2.68	2.69
	EMG	7.44	5.89	5.94	5.41	6.84
	WN	9.95	6.11	5.99	7.47	8.11
	HN	7.27	6.61	5.91	6.85	6.90
-2 dB	ECN	2.59	2.56	2.52	2.55	2.56
	EMG	8.37	5.99	5.84	5.26	6.71
	WN	10.18	5.57	5.03	7.24	7.32
	HN	6.98	6.28	5.34	6.46	6.45
2 dB	ECN	2.84	2.44	2.41	2.44	2.45
	EMG	8.41	5.43	4.12	5.07	6.60
	WN	10.32	3.78	1.93	6.33	5.70
	HN	7.22	5.50	4.22	5.79	5.62
5 dB	ECN	2.80	2.09	2.02	2.09	2.10
	EMG	7.89	4.64	2.57	4.54	5.72
	WN	9.63	4.50	1.83	6.19	5.38
	HN	6.75	4.58	2.75	5.10	4.83
10 dB	ECN	2.56	1.19	1.09	1.21	1.20
	EMG	7.66	3.87	2.05	3.98	4.52
	WN	8.73	2.25	-1.47	4.13	2.66
	HN	6.45	3.89	2.14	4.44	4.11

To verify the similarity degree between noise-free and denoised synthetic ECGs using the 5 denoising methods, the average correlation coefficients of the 6 synthetic ECGs (Syn00-Syn05) are displayed in **Table 7**. According to the data in the table, IEMD-ATD provides the highest average correlation coefficient, which indicates that the denoised ECG from our method has the highest similarity with the original ECG. For EMG, WN and HN at 2 dB, 5 dB and 10 dB, IEMD-ATD provides a correlation coefficient higher than 0.95, and at -5 dB and -2 dB, correlation coefficients higher than 0.75 are given. The correlation coefficients for ECN are the lowest. This is because the frequency range of ECN overlaps with the main frequency of the ECGs.

**Table 7:** Average correlation coefficient of 6 synthetic ECGs (Syn00-Syn05).

Original SNR	Noise Type	Denoising Method				
		IEMD -ATD	UT-H	UT-S	Cust-H	Cust-S
-5 dB	ECN	0.5708	0.5652	0.5475	0.5660	0.5635
	EMG	0.7662	0.6067	0.5795	0.7133	0.7484
	WN	0.8317	0.5834	0.5586	0.7733	0.7380
	HN	0.7715	0.7169	0.6252	0.7555	0.7293
-2 dB	ECN	0.7024	0.7001	0.6878	0.7004	0.6988
	EMG	0.8894	0.7978	0.7793	0.8237	0.8564
	WN	0.9286	0.7598	0.7136	0.8688	0.8437
	HN	0.8600	0.8360	0.7684	0.8484	0.8344
2 dB	ECN	0.8571	0.8468	0.8435	0.8466	0.8463
	EMG	0.9574	0.9104	0.8656	0.9142	0.9345
	WN	0.9712	0.8386	0.7586	0.9278	0.9096
	HN	0.9423	0.9073	0.8705	0.9139	0.9075
5 dB	ECN	0.9217	0.9038	0.9010	0.9038	0.9036
	EMG	0.9750	0.9426	0.9005	0.9481	0.9582
	WN	0.9826	0.9423	0.8872	0.9628	0.9518
	HN	0.9673	0.9347	0.9042	0.9406	0.9366
10 dB	ECN	0.9728	0.9604	0.9594	0.9606	0.9604
	EMG	0.9915	0.9791	0.9664	0.9798	0.9816
	WN	0.9932	0.9662	0.9250	0.9773	0.9701
	HN	0.9888	0.9795	0.9684	0.9819	0.9803

For **Tables 5**, **6** and **7**, the contribution of SNR improvement is not only from high-frequency noise elimination but also from baseline wandering removal. To further inspect the performance of eliminating high-frequency noise, **Table 8** shows the average SNR improvement of the 6 synthetic ECGs (Syn00-Syn05) when only eliminating high-frequency noise with no baseline wandering removal. It is shown that IEMD-ATD is a very effective method for denoising WN and EMG. For HN elimination, IEMD-ATD is still good. For ECN, there is also an improvement for IEMD-ATD, while the denoising of the other methods negatively impacts the SNR, which indicates that hard and soft threshold denoising methods lose useful information when denoising. This provides further proof for the superiority of the proposed peak filtering denoising method in retaining local useful information. In **Table 8**, the SNR improvements for eliminating WN are close to those in **Table 6**. This is because the WN contains no baseline wandering.

**Table 8:** Average SNR improvement of 6 synthetic ECGs (Syn00-Syn05) for eliminating only high-frequency noise.

Original SNR	Noise Type	Denoising Method				
		IEMD -ATD	UT-H	UT-S	Cust-H	Cust-S
-5 dB	ECN	0.03	0.01	-0.01	0.00	0.01
	EMG	0.56	0.25	0.26	0.08	0.44
	WN	9.80	6.18	6.06	7.65	8.33
	HN	0.36	0.22	0.04	0.29	0.29
-2 dB	ECN	0.03	0.01	-0.01	0.00	0.01
	EMG	0.73	0.26	0.21	0.08	0.46
	WN	10.03	5.58	5.03	7.28	7.34
	HN	0.37	0.24	-0.03	0.28	0.27
2 dB	ECN	0.03	0.00	-0.01	0.00	0.01
	EMG	0.77	0.23	-0.23	0.10	0.54
	WN	10.22	4.06	2.12	7.10	6.39
	HN	0.35	0.20	-0.20	0.27	0.23
5 dB	ECN	0.01	0.00	-0.05	0.00	0.00
	EMG	0.78	0.07	-0.78	0.10	0.49
	WN	9.61	4.87	2.01	6.73	5.81
	HN	0.35	0.11	-0.56	0.26	0.19
10 dB	ECN	0.01	-0.01	-0.07	0.00	0.00
	EMG	0.73	0.08	-0.80	0.10	0.30
	WN	9.09	3.79	-0.71	6.26	4.30
	HN	0.30	0.01	-0.78	0.23	0.11

**Table 9** displays the average relative differences of signal energy (ARDE) of 6 synthetic ECGs (Syn00-Syn05). When the correlation coefficient is high, for example, higher than 0.9 (shown in **Table 7**), lower relative differences in signal energy imply that the denoised ECG is more similar to the original noise-free ECG in amplitude. From the table, it can be seen that for 10 dB, 5 dB and 2 dB (except for ECN at 2 dB), the ARDE are within 0.1. EMG, WN and HN are better compared with ECN. When the noise is large, e.g., ARDE of -5 dB and -2 dB, the ARDE is higher than 0.1, which indicates a large difference in the energy between the denoised ECG and the original noise-free ECG. It is worth noting that a negative ARDE indicates information loss in the denoised ECG, while a positive ARDE indicates remaining noise.

**Table 9:** Average relative differences in signal energy of 6 synthetic ECGs (Syn00-Syn05).

Original SNR	Noise Type	Denoising Method				
		IEMD -ATD	UT-H	UT-S	Cust-H	Cust-S
-5 dB	ECN	1.449	1.506	1.431	1.515	1.491
	EMG	0.377	0.136	-0.015	0.856	0.500
	WN	-0.042	-0.357	-0.382	0.395	-0.203
	HN	0.463	0.387	0.185	0.523	0.336
-2 dB	ECN	0.721	0.724	0.670	0.729	0.713
	EMG	0.110	0.109	-0.006	0.467	0.273
	WN	0.097	-0.144	-0.230	0.214	-0.123
	HN	0.207	0.224	0.019	0.275	0.149
2 dB	ECN	0.227	0.266	0.245	0.265	0.260
	EMG	0.083	0.033	-0.119	0.193	0.076
	WN	0.037	-0.157	-0.352	0.081	-0.187
	HN	0.053	0.042	-0.104	0.062	-0.014
5 dB	ECN	0.091	0.090	0.067	0.089	0.083
	EMG	0.039	0.009	-0.111	0.106	0.032
	WN	0.015	-0.017	-0.200	0.054	-0.102
	HN	0.017	-0.015	-0.153	0.003	-0.057
10 dB	ECN	0.015	0.021	0.009	0.021	0.018
	EMG	0.006	0.022	-0.047	0.034	0.011
	WN	0.005	-0.034	-0.193	0.001	-0.100
	HN	-0.004	0.001	-0.075	0.005	-0.025

The whole procedure includes ECG decomposition, IMF grouping, IMF denoising and ECG reconstruction. The relative computation time of ECG decomposition is displayed in **Table 1**. **Table 10** shows the computation times of IMF grouping, denoising all the IMFs of one ECG signal and reconstruction. The computing environment is MATLAB 2016 using Windows 10 x64. RAM: 4 GB, CPU: Intel Core i5 2.8 GHz.

**Table 10:** Computation time.

Grouping	Denoising (the proposed)	Denoising (the others)	Reconstructing
0.015 s	0.1 s	0.001 s	negligible

From the table above, we can conclude that the time cost of grouping, denoising and reconstruction is acceptable.

#### 4.2.2 Results for real data

Similar experiments to those performed for synthetic data are carried out for real data in this section. The red lines represent the original noise-free real ECGs. The blue line represents the noisy ECGs. The black lines represent the denoised ECGs by IEMD-ATD and the corresponding EMD denoising methods.

**Fig 13:**
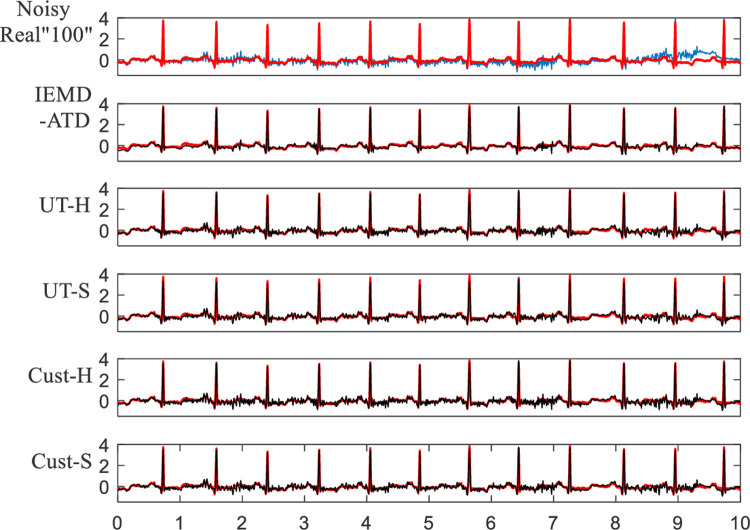
Waveforms of denoised ECGs for real “100” with EMG noise at 5 dB for the 5 denoising methods.

**Fig 14:**
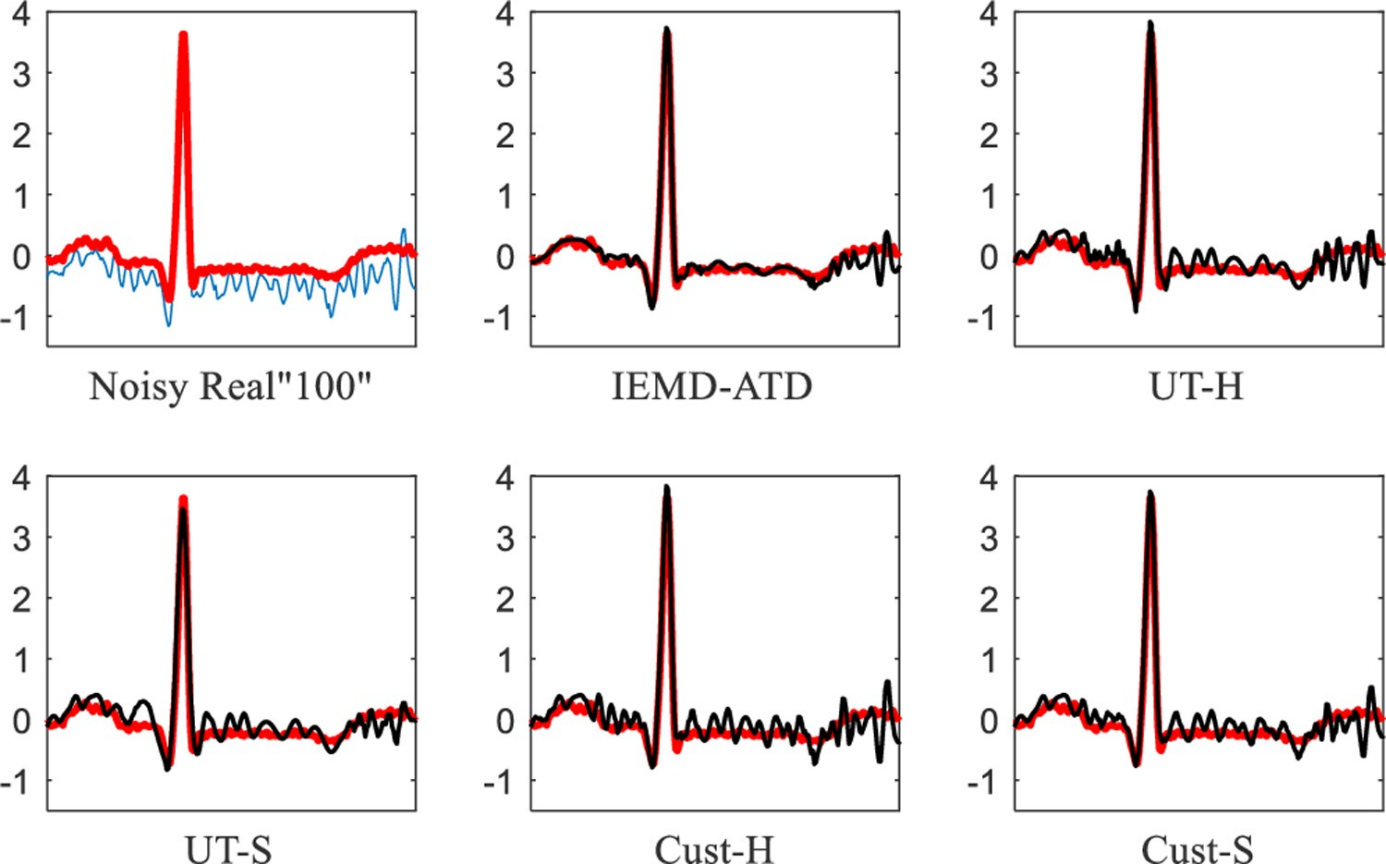
Details of waveforms between 6.2 and 6.85 seconds from Fig 13.

In **Fig 13**, Real “100” with 5 dB EMG noise is used for denoising experiments. It can be seen that IEMD-ATD provides the “smoothest” waveform of denoised ECGs with the least noise remaining compared with the other 4 methods. In addition, for the noisy Real “100” (the first curve in **Fig 13**), there is an obvious baseline wandering; it is eliminated effectively by the denoising process. **Fig 14** depicts the local QRS waveform details in the denoised ECGs between 6.2 and 6.85 seconds. This indicates that the proposed method not only eliminates the noise effectively but also restores and preserves the QRS waveforms. Compared with our method, the other methods leave more noise and unclean Q and S waves.

**Fig 15:**
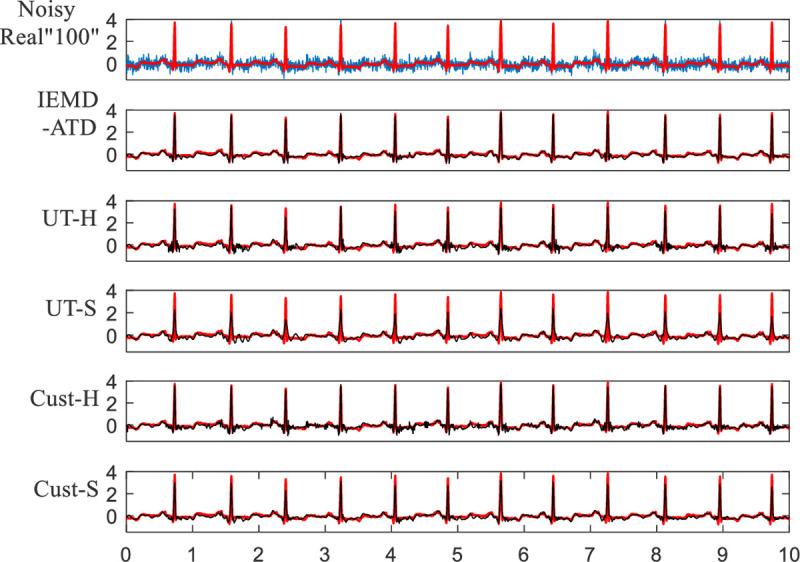
Waveforms of denoised ECG for real “100” with WN at 5 dB for the 5 denoising methods.

**Fig 16:**
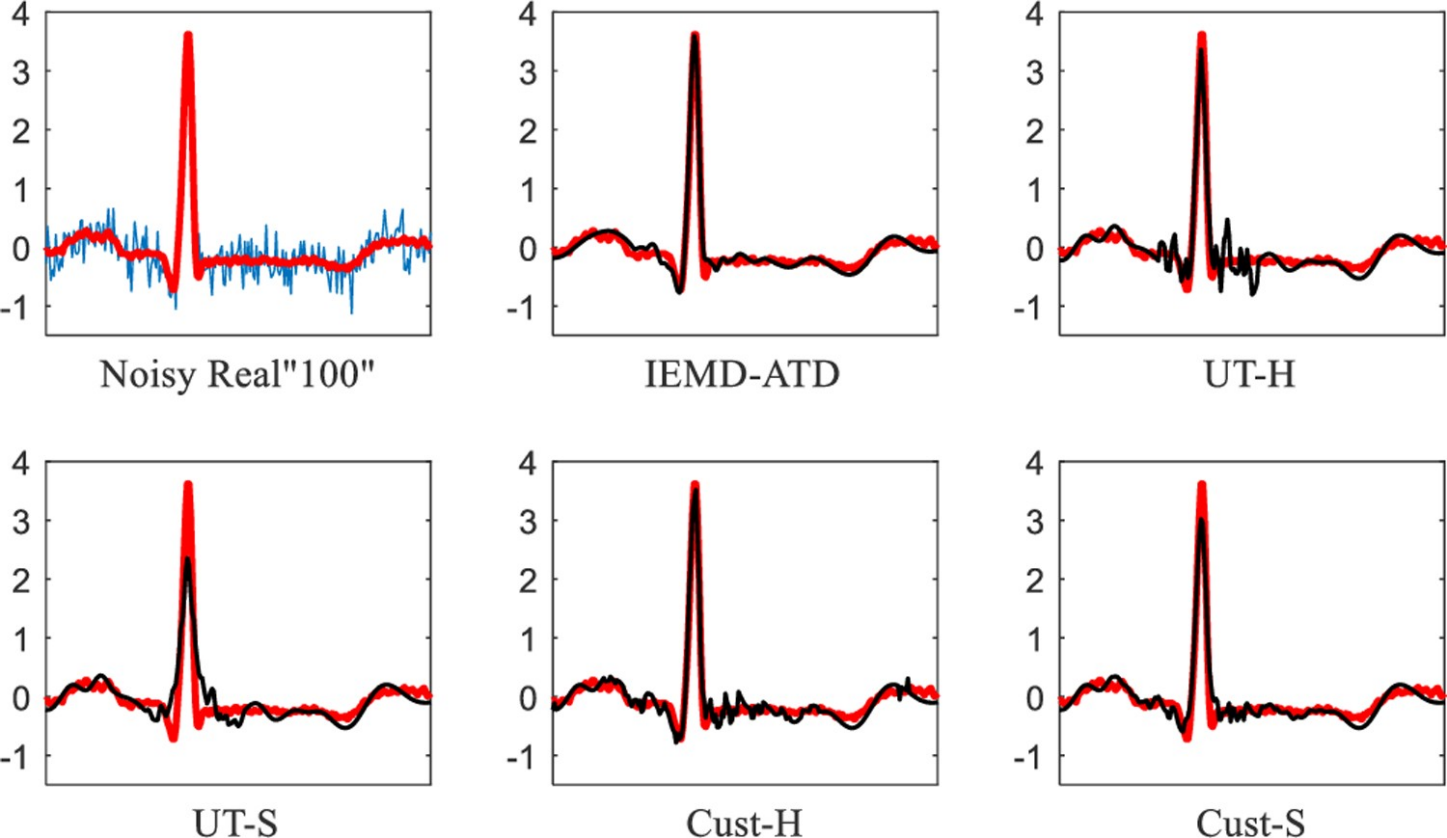
Details of waveforms between 6.2 and 6.85 seconds from Fig 15.

**Fig 15** depicts the denoising waveforms of Real “100” with WN at 5 dB. As the figure suggests, IEMD-ATD has the best performance with a “smooth” and clean denoised ECG, which has the highest coincidence degree. **Fig 16** shows the local waveform details of the denoised Real “100” with WN at 5 dB between 6.2 and 6.85 seconds. It can be seen that UT-S and Cust-S have a large loss of amplitude of R peaks and a larger waveform distortion of Q and S waves. For UT-H and Cust-H, considerable noise remains around the Q and S waves. From the figure, IEMD-ATD offers the best denoising performance for cleaner, clearer and more intact QRS waveforms.

SNR improvements of the 5 denoising methods for 10 real ECGs are displayed in **Table 11**. The original SNR of the noisy ECG is 5 dB. The noises used here are EMG and WN. “Avg” in the table represents the average SNR improvement of 10 real ECG signals. According to **Table 11**, the denoising performance of IEMD-ATD is good, with almost all SNR improvements being higher than 4 dB except for Real “117”. However, the other 4 methods cannot provide effective denoising results for Real “117”.

**Table 11:** SNR improvement of 5 denoising methods for real ECGs contaminated with EMG and WN at 5 dB.

Noise Type	ECG (Real)	Denoising Method				
		IEMD–ATD	UT-H	UT-S	Cust-H	Cust-S
EMG	100	7.76	4.79	4.25	4.27	4.81
	101	3.84	1.48	0.80	1.27	1.69
	103	5.06	3.47	1.87	3.18	4.04
	112	4.81	2.94	3.03	2.38	2.77
	113	6.59	1.88	0.57	1.48	2.00
	115	5.73	1.41	1.29	1.15	1.42
	117	0.12	0.62	0.14	0.15	0.48
	119	7.09	4.50	3.36	4.15	4.92
	122	6.60	5.50	5.70	4.48	5.35
	123	4.25	2.34	-0.27	2.59	3.30
	Avg	5.18	2.89	2.07	2.51	3.08
WN	100	8.12	2.84	-0.97	5.76	3.88
	101	4.35	0.65	-1.13	2.02	1.67
	103	5.70	0.30	-1.66	3.16	2.09
	112	5.79	2.32	1.16	4.21	4.12
	113	6.46	-3.54	-4.05	2.23	1.86
	115	6.49	2.82	-0.04	3.93	3.17
	117	-0.06	0.30	-0.98	0.83	0.74
	119	9.01	2.30	-0.08	5.05	4.11
	122	5.51	2.81	2.23	5.43	5.12
	123	6.38	3.28	-0.60	4.52	3.78
	Avg	5.78	1.41	-0.61	3.71	3.05

**Table 12** displays the average SNR improvement of 10 real ECGs with the original SNRs of -5 dB, -2 dB, 2 dB, 5 dB and 10 dB. The results indicate that our proposed method has the best performance for ECG denoising. According to the data, the effects of eliminating EMG, WN and HN by our method are prominently better than those of the other methods.

**Table 12:** Average SNR improvement of 10 real ECGs.

Original SNR	Noise Type	Denoising Method				
		IEMD -ATD	UT-H	UT-S	Cust-H	Cust-S
-5 dB	ECN	2.79	2.65	2.61	2.63	2.65
	EMG	7.13	5.65	5.86	5.12	6.13
	WN	9.61	6.63	6.54	7.36	8.21
	HN	7.09	6.13	5.63	6.53	6.69
-2 dB	ECN	2.58	2.32	2.25	2.32	2.34
	EMG	7.58	5.17	5.32	4.79	5.59
	WN	8.77	4.70	4.35	6.64	6.80
	HN	6.75	5.74	4.89	6.06	6.09
2 dB	ECN	2.27	1.82	1.65	1.84	1.85
	EMG	6.66	4.40	4.16	3.70	4.46
	WN	7.26	2.60	1.44	5.07	4.56
	HN	5.82	4.79	3.53	5.14	5.03
5 dB	ECN	1.69	1.49	1.17	1.57	1.57
	EMG	5.18	2.89	2.07	2.51	3.08
	WN	5.78	1.41	-0.61	3.71	3.05
	HN	4.77	3.15	2.02	3.45	3.34
10 dB	ECN	0.40	-0.78	-1.25	-0.68	-0.71
	EMG	2.83	0.42	-1.21	0.48	0.82
	WN	3.19	-0.09	-2.41	0.89	0.24
	HN	2.48	0.76	-1.06	1.47	1.23

The average correlation coefficient between noise-free and denoised real ECGs (10 signals) by the 5 denoising methods is displayed in **Table 13**. According to the data in the table, IEMD-ATD provides the highest average correlation coefficient compared with the other 4 methods, which indicates that IEMD-ATD provides a denoised ECG with a high similarity to the original ECG.

**Table 13:** Average correlation coefficient of 10 real ECGs.

Original SNR	Noise Type	Denoising Method				
		IEMD -ATD	UT-H	UT-S	Cust-H	Cust-S
-5 dB	ECN	0.5891	0.5843	0.5634	0.5882	0.5848
	EMG	0.7024	0.6357	0.6391	0.6778	0.7071
	WN	0.8237	0.5856	0.5705	0.7542	0.7460
	HN	0.7675	0.6722	0.6010	0.7392	0.7190
-2 dB	ECN	0.7109	0.6887	0.6681	0.6905	0.6878
	EMG	0.8639	0.7520	0.7466	0.7879	0.8098
	WN	0.8942	0.6892	0.6532	0.8328	0.8155
	HN	0.8528	0.8087	0.7460	0.8315	0.8193
2 dB	ECN	0.8383	0.8265	0.8108	0.8281	0.8266
	EMG	0.9265	0.8771	0.8672	0.8656	0.8807
	WN	0.9325	0.7939	0.7290	0.8982	0.8818
	HN	0.9161	0.8950	0.8487	0.9051	0.8986
5 dB	ECN	0.8944	0.8933	0.8809	0.8954	0.8944
	EMG	0.9433	0.9116	0.8871	0.9066	0.9154
	WN	0.9481	0.8545	0.7730	0.9268	0.9153
	HN	0.9397	0.9140	0.8851	0.9197	0.9165
10 dB	ECN	0.9525	0.9362	0.9281	0.9376	0.9369
	EMG	0.9703	0.9443	0.9187	0.9452	0.9481
	WN	0.9710	0.9431	0.9045	0.9537	0.9475
	HN	0.9685	0.9487	0.9268	0.9554	0.9533

Similar to the synthetic ECG experiments, to inspect the performance of further eliminating high-frequency noise, the average SNR improvement of 10 real ECGs for eliminating only high-frequency noise without baseline wandering removal is displayed in **Table 14**. For WN denoising, since there are no baseline wandering components in WN that are generated through MATLAB, there are increased SNR improvements. The proposed IEMD-ATD has an obviously better performance for eliminating WN with increased SNR improvements compared with the other methods. This implies that the peak filtering denoising method is better than the hard and soft denoising methods when eliminating high-frequency noise. For EMG, our method is also the best denoising strategy compared with the other methods. It is worth noting that when the original SNR is high, many negative SNR improvements appear, especially in ECN elimination. This is because ECN has a fluctuation frequency more similar to that of ECG signals. This may lead to errors when eliminating noise using the threshold methods. When the original SNR is higher, which means less noise, it is prone to removing ECG values rather than noise values. As a whole, the proposed method is superior to the other 4 methods in improving SNR.

**Table 14:** Average SNR improvement of 10 real ECGs for eliminating only high-frequency noise.

Original SNR	Noise Type	Denoising Method				
		IEMD -ATD	UT-H	UT-S	Cust-H	Cust-S
-5 dB	ECN	0.03	0.01	-0.01	0.00	0.01
	EMG	0.67	0.21	0.27	0.06	0.33
	WN	10.15	6.91	6.83	7.66	8.59
	HN	0.38	0.18	0.04	0.29	0.32
-2 dB	ECN	0.03	0.00	-0.04	0.00	0.01
	EMG	0.76	0.14	0.17	0.04	0.29
	WN	9.79	5.36	4.95	7.56	7.77
	HN	0.37	0.18	-0.08	0.28	0.28
2 dB	ECN	0.03	-0.01	-0.12	0.00	0.01
	EMG	0.81	0.33	0.23	0.03	0.33
	WN	9.61	3.94	2.45	7.06	6.39
	HN	0.35	0.16	-0.33	0.27	0.24
5 dB	ECN	0.02	-0.05	-0.27	0.00	0.00
	EMG	0.79	0.25	-0.26	0.02	0.33
	WN	9.29	3.11	0.37	6.82	5.66
	HN	0.32	0.11	-0.51	0.24	0.20
10 dB	ECN	-0.05	-0.14	-0.65	-0.02	-0.05
	EMG	0.68	-0.01	-1.48	0.01	0.30
	WN	8.62	3.60	-0.53	5.99	4.37
	HN	0.23	-0.31	-1.63	0.19	0.05

**Table 15** displays the average relative differences of signal energy (ARDE) of 10 real ECGs. From the table, similar to the results in synthetic ECG, for 10 dB, 5 dB and 2 dB (except for ECN at 2 dB), the ARDE are within 0.1. EMG, and WN and HN are better compared with ECN.

**Table 15:** Average relative differences in signal energy of 10 real ECGs.

Original SNR	Noise Type	Denoising Method				
		IEMD -ATD	UT-H	UT-S	Cust-H	Cust-S
-5 dB	ECN	1.545	1.614	1.532	1.645	1.613
	EMG	0.137	0.362	0.274	0.797	0.542
	WN	-0.002	-0.278	-0.294	0.315	-0.187
	HN	0.497	0.325	0.191	0.539	0.352
-2 dB	ECN	0.764	0.758	0.678	0.771	0.748
	EMG	0.072	0.125	0.068	0.383	0.264
	WN	0.030	-0.326	-0.374	0.101	-0.261
	HN	0.218	0.185	0.030	0.265	0.145
2 dB	ECN	0.248	0.303	0.233	0.311	0.294
	EMG	-0.010	-0.020	-0.086	0.095	0.029
	WN	-0.060	-0.228	-0.340	0.014	-0.224
	HN	0.036	0.042	-0.101	0.072	-0.004
5 dB	ECN	0.074	0.104	0.042	0.110	0.095
	EMG	-0.054	-0.053	-0.155	0.013	-0.032
	WN	-0.090	-0.203	-0.372	-0.032	-0.219
	HN	-0.031	-0.025	-0.146	-0.012	-0.064
10 dB	ECN	-0.010	-0.007	-0.062	-0.005	-0.018
	EMG	-0.055	-0.066	-0.171	-0.038	-0.062
	WN	-0.067	-0.078	-0.209	-0.043	-0.133
	HN	-0.048	-0.053	-0.154	-0.040	-0.077

## 5. Conclusion

In this paper, an integrated EMD adaptive threshold denoising method (IEMD-ATD) is proposed for processing ECG signals. IEMD-ATD consists of a new method for grouping the IMFs of ECG signals, an adaptive threshold determination method based on the 3σ criterion and a peak filtering denoising method. The experimental results of both synthetic and real ECGs in MIT-BIT show that the IEMD-ATD proposed in this paper offers good performance on IMF grouping and baseline wandering elimination for ECG signals. The adaptive threshold determined is more reasonable, and the effect of filtering high-frequency noise is better than the existing EMD hard and soft threshold denoising methods. The SNR is improved significantly, and the waveform of the QRS complex remains more intact. IEMD-ATD solves the problems of local distortion and discontinuity caused by hard and soft threshold denoising methods. IEMD-ATD is simple in calculation, strong in adaptability and wide in application. It offers great advantages in denoising ECG signals.
